# Correction: Raising awareness of potential biases in medical machine learning: Experience from a Datathon

**DOI:** 10.1371/journal.pdig.0001616

**Published:** 2026-07-27

**Authors:** Harry Hochheiser, Jesse Klug, Thomas Mathie, Tom J. Pollard, Jesse D. Raffa, Stephanie L. Ballard, Evamarie A. Conrad, Smitha Edakalavan, Allan Joseph, Nader Alnomasy, Sarah Nutman, Veronika Hill, Sumit Kapoor, Eddie Pérez Claudio, Olga V. Kravchenko, Ruoting Li, Mehdi Nourelahi, Jenny Diaz, W. Michael Taylor, Sydney R. Rooney, Maeve Woeltje, Leo Anthony Celi, Christopher M. Horvat

The first author, Harry Hochheiser, is incorrectly noted as the corresponding author. The correct corresponding author is Jesse D. Raffa. Jesse D. Raffa’s email address is: jraffa@mit.edu.
