# Supplementary material for: Raising awareness of potential biases in medical machine learning: Experience from a Datathon
Source: PLOS Digit Health. 2025 Jul 11;4(7):e0000932. doi: 10.1371/journal.pdig.0000932 (PMC12250157; doi:10.1371/journal.pdig.0000932)
Supplement: Attachment — Submitted filename: PDIG-D-24-00484.docx [file pdig.0000932.s001.docx]

Title: Raising awareness of potential biases in medical machine learning: Experience from a Datathon

Thank you for the opportunity to review this interesting piece of work. Find my comments hereunder.

Overview

The paper addresses the critical issue of potential biases in medical ML by enhancing users’ awareness of such issues through participation in a datathon. While AI is helpful in healthcare, there are still issues that need to be addressed, such as bias, which is often hidden in the *blackbox* nature of AI models, hence, the rise of XAI in healthcare. Thus, identifying and letting people know of biases is an important endeavour. However, the paper needs substantial improvements. For example, there is no literature review, which is critical in any research paper.

Introduction

Bias is a huge issue, and the introduction could be expanded to provide more context and a strong argument for bias avoidance and awareness.

Literature review

Provide a clearly delineated literature review. While it is possible to not have a dedicated section on literature review or related works following the IMRaD structure, the introduction should be detailed and long enough to provide a review of the literature, which isn’t the case in this manuscript.

Materials and methods

This section needs to be revised. E.g. The statement “Although logistic regression models based on demographics, labs, vitals, and APACHE score predictions led to models with good performance and calibration [7], potential biases in the data have not yet been thoroughly studied” is more of a research gap than methodology, especially the part “..potential biases in the data have not yet been thoroughly studied”. Describe how the submissions were analysed.

Results

The statement “Sixteen participants were assigned into five teams of equal size …” needs to be looked into, as 16 is indivisible by 5, hence, the teams can’t be of equal size. Again, if there were 5 equal teams out of 16 participants, team sizes cannot be 5, hence the statement “…final team sizes ranged from 1 to 5 members” needs to be corrected.

This part is more of methodology than results, as it describe ‘how’ the datathon was conducted:

“Of the five teams, two used the synthetic UPMC dataset, two used the women in science dataset, and one team used both. No teams used the eICU-CRD dataset Python was the preferred analysis tool, selected by four of the five teams. The remaining team used R. At least one team used a GUI-based tool for exploratory data analysis, and one team acknowledged using ChatGPT to assist with coding”

Discussion

Table 2 might have been misplaced, as it is cross-referenced in results but placed under the discussion section.
