# Supplementary material for: Raising awareness of potential biases in medical machine learning: Experience from a Datathon
Source: PLOS Digit Health. 2025 Jul 11;4(7):e0000932. doi: 10.1371/journal.pdig.0000932 (PMC12250157; doi:10.1371/journal.pdig.0000932)
Supplement: Attachment — Submitted filename: PDIG-D-24-00484 R1.docx [file pdig.0000932.s002.docx]

Title: Raising awareness of potential biases in medical machine learning: Experience from a Datathon R1

Thank you for revising the manuscript. The paper looks way better than the first version. However, a few things may need to be attended to. Find my comments hereunder.

Author summary

Assuming the editor finds it befitting to retain it, perhaps you could enhance the first sentence by stating examples of the ‘disadvantaged groups’ that are at risk by including a ‘such as ….’.

Overview

Thanks for significantly improving your manuscript and responding to all the reviewer comments.

Introduction

After striking out the opening sentence that introduced the full form of ML, ensure that you expand the abbreviation before using it. Also, expand AI on first use, and every other acronym/abbreviation.

The sentence “Inappropriate biases in machine learning models arise when models offer predictions or classifications based on an individuals’ identity, rather than their medical condition” may need to be substantiated. Also, “inappropriate biases” may be construed to mean some biases are ‘appropriate’, yet the term ‘bias’ inherently has a negative connotation. While there may be some ‘necessary biases’, such as risk stratification, it would perhaps help readers if the authors expand on this, including explicitly stating that some risks may be appropriate, along with giving examples and those of inappropriate biases. Otherwise, remove the word ‘inappropriate’.

Proofread the work again to eliminate obvious errors, such as “predicto” and other typos.

This claim may need to be substantiated: “These Efforts are complicated by the absence of clear definitions of bias or related terms such as model fairness”

Discussion

In the authors’ response to Reviewer Point P 1.2, they state that “In the discussion section, we have carefully aligned our conclusions with the results presented, ensuring clear and justified interpretations throughout the manuscript.” However, I do not see any marked changes in the discussion section.
